# P67 - Mothers restrict physical activity for children and teens asthmatics

**DOI:** 10.1186/2045-7022-4-S1-P122

**Published:** 2014-02-28

**Authors:** Marco Correia Junior, Fabianne Assis, Décio Medeiros, José Angelo Rizzo, Silvia Sarinho, Emanuel Sarinho

**Affiliations:** 1 University of Pernambuco, Petrolina, Brazil; 2 Federal University of Pernambuco, Recife, Brazil

## Background

Physical activities (PA) are important for children and adolescents, especially in asthmatics. A significant proportion of them are considered less active than their non-asthmatic peers and maternal health beliefs have been pointed as determinant factor.

## Aims and objectives

To investigate if mothers impose limitations on physical activities of their asthmatic children, try to identify associated factors and ascertain if this attitude affects activities level.

## Methods

In this cross sectional study were included 115 asthmatic children and adolescents 9 to 19 years old and their mothers. For the children, asthma severity, PA level and exercise induced bronchospasm (EIB) were evaluated. The mothers were investigated on their beliefs about PA in non-asthmatic and asthmatic children, if they imposed restrictions to their children PA, perception of exercise induced asthma and with an anxiety and depression questionnaire.

## Results

Almost all the mothers reported that PA are important for children and adolescents. Despite this, 37% (43/115) of them admitted that they imposed restrictions to their children PA. This attitude was associated with asthma severity and to maternal factors such as dyspnea perception after treadmill running, negative opinions about PA in asthmatics and anxiety. Despite this, their children were not less active than those from non restrictive mothers (odds ratio, 0.89; 95% confidence interval, 0.41–1.95).

## Conclusions

A considerable proportion of mothers report to impose limitations on their children PA. Parental and caregiver beliefs and fears should be discussed about in order to avoid conflicts and negative attitudes that could discourage their children taking part in physical activities and sports.

